# Marine Incursion: The Freshwater Herring of Lake Tanganyika Are the Product of a Marine Invasion into West Africa

**DOI:** 10.1371/journal.pone.0001979

**Published:** 2008-04-23

**Authors:** Anthony B. Wilson, Guy G. Teugels, Axel Meyer

**Affiliations:** 1 Department of Biology, University of Konstanz, Konstanz, Germany; 2 Zoological Museum, University of Zurich, Zurich, Switzerland; 3 Ichthyology Laboratory, Royal Museum for Central Africa, Tervuren, Belgium; University of California, Berkeley, United States of America

## Abstract

The spectacular marine-like diversity of the endemic fauna of Lake Tanganyika, the oldest of the African Great Lakes, led early researchers to suggest that the lake must have once been connected to the ocean. Recent geophysical reconstructions clearly indicate that Lake Tanganyika formed by rifting in the African subcontinent and was never directly linked to the sea. Although the Lake has a high proportion of specialized endemics, the absence of close relatives outside Tanganyika has complicated phylogeographic reconstructions of the timing of lake colonization and intralacustrine diversification. The freshwater herring of Lake Tanganyika are members of a large group of pellonuline herring found in western and southern Africa, offering one of the best opportunities to trace the evolutionary history of members of Tanganyika's biota. Molecular phylogenetic reconstructions indicate that herring colonized West Africa 25–50MYA, at the end of a major marine incursion in the region. Pellonuline herring subsequently experienced an evolutionary radiation in West Africa, spreading across the continent and reaching East Africa's Lake Tanganyika during its early formation. While Lake Tanganyika has never been directly connected with the sea, the endemic freshwater herring of the lake are the descendents of an ancient marine incursion, a scenario which may also explain the origin of other Tanganyikan endemics.

## Introduction

Ancient lakes are home to disproportionate levels of freshwater biodiversity. As standing bodies of water which have existed for at least 100,000 years [1], these habitats have been remarkably stable when compared to more typically transitory freshwater environments. As a consequence, lakes such as Lake Baikal (25–30 MY) and the African Great Lakes Malawi (1–2 MY) and Tanganyika (9–12 MY) all contain exceptionally high numbers of freshwater taxa, of which up to 99% are endemics [2].

Habitat stability is thought to promote niche partitioning and resource specialization, providing an important engine for speciation [3]. Due to the relative stability of ancient lakes, these habitats have long been recognized as centers of spectacular adaptive radiation, exemplified by the highly specialized cichlid fishes of the African Great Lakes [4], [5]. This pattern of often rapid *in situ* adaptive radiation has also been documented in other groups of fishes [6] and invertebrates [7]–[9]. At the same time, there is a growing appreciation that in addition to their role as centers of diversification, ancient lakes have also played an important role as evolutionary reservoirs, maintaining diverse groups of organisms that have been extirpated outside their borders [10]–[13].

While each of the African Great Lakes is home to high levels of endemic biodiversity, Lake Tanganyika is distinguished from Lakes Malawi and Victoria by the taxonomic breadth and morphological diversity of its endemic fauna. Freshwater lineages of crustaceans and gastropods found in the lake exhibit striking morphological similarities to marine species and cnidarians and clupeiform fishes, groups typically restricted to marine environments, are also found within the lake [14]. Due to strong morphological affinities between Lake Tanganyika's fauna and marine organisms, early investigators proposed that the lake must have been directly connected to the ocean at some point in its history [15], [16]. Subsequent investigations of the geology of the region however indicate that the African Great Lakes were formed by rifting in the African subcontinent and were thus never in direct contact with the ocean [17], [18]. While the hypothesis of a direct marine connection [16] appears invalid, the enigma of the “Tanganyika Problem” remains unanswered: namely, how did such a specialized and unique freshwater biota come to be found within the Lake?

Unfortunately, attempts to elucidate the evolutionary origins of Tanganyikan endemics have been hampered by the absence of close relatives outside the lake. While Tanganyika appears to have been colonized by at least four ancient lineages of gastropods [12] and eight seeding lineages of cichlid fishes [11], the colonization history of these groups cannot be easily traced due to the absence of close extant and/or fossil relatives in the African subcontinent.

While clupeid fishes dominate marine fish communities and anadromous populations inhabit brackish waters, freshwater diversity of this group is typically low. West Africa is home to the largest evolutionary radiation of freshwater clupeid fishes [19], including at least twenty species of the subfamily Pellonulinae. The pellonuline herring of West Africa exhibit striking adaptations for life in freshwater, including carnivorous forms with large canine teeth (*Cyanothrissa* and *Odaxothrissa*), species almost completely lacking scales (*Thrattidion*) and a general tendency towards reduced size, exemplified by species that attain sexual maturity at less than 20 mm SL (*Thrattidion* and *Sierrathrissa*) [20]. Pellonuline herring are also found in southern and central Africa and Madagascar as well as Australia, India and eastern Asia [21]. Two pellonuline species are endemic to Lake Tanganyika, where they are dominant members of the pelagic zone [22].

A comprehensive treatment of fossil and recent clupeomorph fishes has questioned the monophyly of clupeid subfamilies, including the pellonulines [21]. The pellonuline herring of Africa appear to fall into two major groups, the Pellonulini, a tribe containing taxa from western and central Africa, and the Ehiravini, a tribe of herring from southern Africa and India [21]. A more recent morphological investigation of African pellonuline herring supported this hypothesis and suggested that the herring of Lake Tanganyika are closely allied with those of West Africa [19]. A clear understanding of the historical biogeography of pellonuline herring may be one of our best opportunities to reconstruct the evolutionary history of members of the unique fauna of Lake Tanganyika.

**Figure 1:**
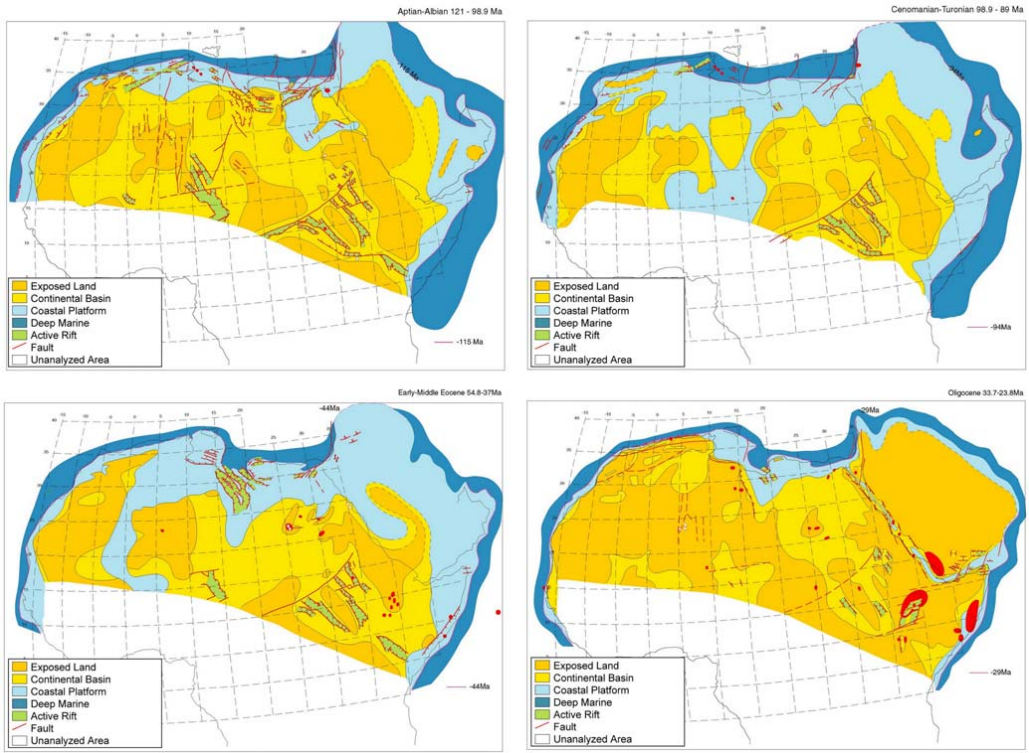
Paleotectonic reconstruction of the African subcontinent illustrating a major marine incursion in the African subcontinent which lasted from the late Cretaceous (Cenomanian-Turonian) through the end of the Eocene (Early-Middle Eocene). Figures adapted with permission from the author [74].

Here, we construct a phylogeny of clupeiform fishes based on three mitochondrial DNA genes and use a multipoint fossil calibration to determine both the timing of freshwater colonization of Africa by pellonuline herring and the timing of the colonization and diversification of herring within Lake Tanganyika. Molecular phylogenetic reconstructions reject the monophyly of pellonuline herring and support strong affinities between the endemic herring of Tanganyika and freshwater pellonulines found in western Africa. Molecular clock analyses indicate that the colonization of African freshwater by marine herring occurred during the Eocene (25–50 MYA), at the end of a period of major marine incursion in West Africa [Fig. 1; 23]. Herring subsequently spread across central Africa, colonizing Lake Tanganyika and diversifying into the two present-day endemics 2–16 MYA.

## Results

### Preliminary Sequence Analyses

Mitochondrial sequences of 12S rDNA, 16S rDNA and cytochrome *b* (Cyt*b*) were collected, collated and aligned for 49 species (90 specimens), resulting in a total sequence length of between 1,360 and 2,510 bp per specimen. Despite repeated attempts to amplify Cyt*b* from *Spratelloides robustus* (H101), this individual failed to yield any PCR product for this gene. While a ML homogeneity test rejected congruency of sequences from the three target loci (p<0.003 for all topologies), sequence data were concatenated in accordance with a total evidence approach [24]. Analyses of Cyt*b* sequence data revealed saturation of third codon transitions for Kimura-2-parameter distances greater than 0.40, a pattern confirmed by a statistical test [25] which indicated substantial saturation at third codon positions (Iss<Iss.sym; p = 0.388). Third codon positions of Cyt*b* were consequently eliminated from further analyses, resulting in a concatenated dataset of between 1,049 and 1,811 bp of sequence data per individual.

### Phylogenetic Relationships among Clupeiform Fishes

**Figure 2:**
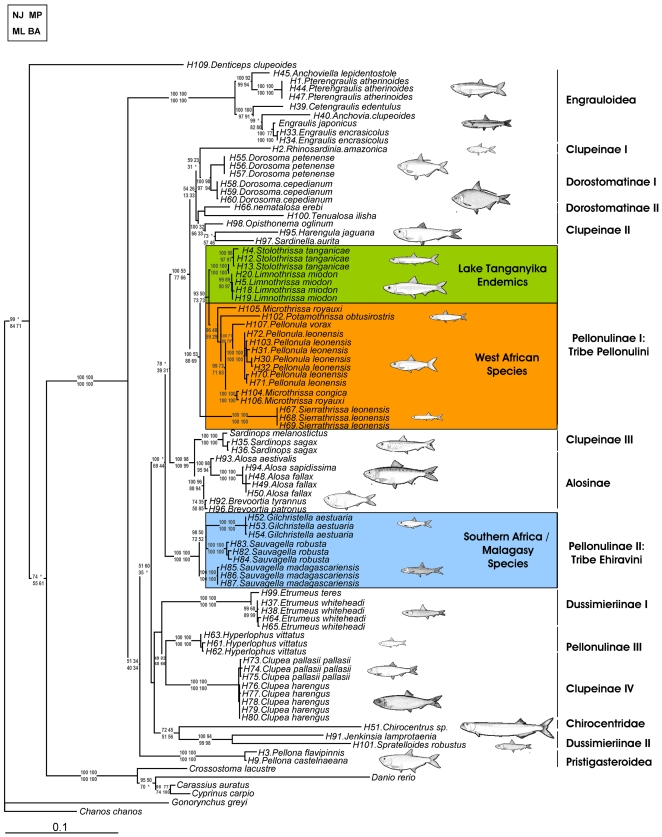
Maximum likelihood tree topology based on the combined dataset of 1,811bp of 12S, 16S and Cytochrome b. Numbers on branches represent bootstrap support for Distance, Maximum Parsimony and Maximum Likelihood analyses and posterior probabilities from Bayesian analysis. Traditional Clupeoid groups and major African freshwater lineages indicated. Clupeid diagrams reprinted with the permission of the Food and Agriculture Organization of the United Nations [34], [75].

While molecular phylogenetic reconstruction provided strong support for most subfamily groups of clupeiforms, resolution was weaker at deeper levels of the phylogeny (Fig. 2). Nonetheless, several major patterns were clear. Molecular phylogenetic analyses uncovered most traditional groupings of clupeiform fishes (Fig. 2) and identified several major incongruencies with previous morphological-based phylogenies [reviewed in 21]. Although the maximum likelihood phylogeny placed *Denticeps clupeoides*, the sole living member of the Denticipitoidei, outside the Clupeiformes (Fig. 2), a Shimodaira-Hasegawa (SH) test did not reject a monophyletic clupeiform assemblage (Table 1; LRT: P = 0.8963). Phylogenetic reconstruction supported the monophyly of the Engrauloidea and Pristigasteroidea, along with the Chirocentridae and Alosinae. In contrast, the Clupeinae, Pellonulinae, Dorostomatinae and Dussimierinae all formed polyphyletic assemblages (Fig. 2) and monophyly could be statistically rejected for both the Clupeinae and Pellonulinae (Table 1; SH LRT test: p<0.001 for both subfamilies).

**Table 1:** Shimodiara-Hasegawa [63] test of alternative topologies.

Topology	Likelihood	L_max_-L_α_	*P*
ML Topology (Fig. 2)	14940.81	0.0	
Monophyletic Clupeiformes (Denticipitoidei, Clupeoididei)	14941.77	0.96	0.8963
Monophyletic Clupeidae (Dussumieriinae, Pellonulinae, Dorosomatinae, Clupeinae, Alosinae)	14945.02	4.21	0.8049
Monophyletic Dorostomatinae	14946.51	5.70	0.7103
Monophyletic Dussumieriinae	14950.11	9.30	0.6529
Monophyletic Clupeinae	15085.89	145.08	<0.001
Monophyletic Pellonulinae	15021.73	80.92	<0.001

Tree topology, estimated likelihood, log-likelihood differences and *P*-values for alternative topologies tested (χ^2^-test). L_max_: Maximum likelihood topology; L_α_: Likelihood of topology α.

### Polyphyly of African Pellonuline Herring

The pellonuline herring of Africa fall into two major lineages, consistent with Grande's [21] suggested taxonomic groupings (Fig. 2). The first group (tribe Ehiravini) includes *Sauvagella* spp. and *Gilchristella aestuaria*, riverine herring from southern Africa and Madagascar [26]. The second group (tribe Pellonulini) contains *Limnothrissa miodon* and *Stolothrissa tanganicae*, the two species restricted to Lake Tanganyika, and a large group of West African herring. *Hyperlophus vittatus*, an Australian pellonuline, forms part of a third cluster of non-pellonuline herring (Fig. 2). As highlighted above, a SH test rejected the monophyly of the Pellonulinae (Table 1; LRT: P<0.001). The herring of Tanganyika are nested within a larger group of West African herring (Fig. 2) and are most parsimoniously derived from this group (West Africa→Tanganyika: 1 Step; Tanganyika→West Africa: 2 Steps).

### Local and Relaxed Molecular Clocks

A global molecular clock was rejected in favor of lineage-specific rates of molecular evolution (LRT: unconstrained model –ln L = 14940.81; constrained model –ln L = 15209.60; χ^2^_88_ = 537.58; p<0.001). A Bayesian-based approach, incorporating multiple fossil calibration points, was used to estimate divergence times for critical nodes in the phylogeny.

**Figure 3:**
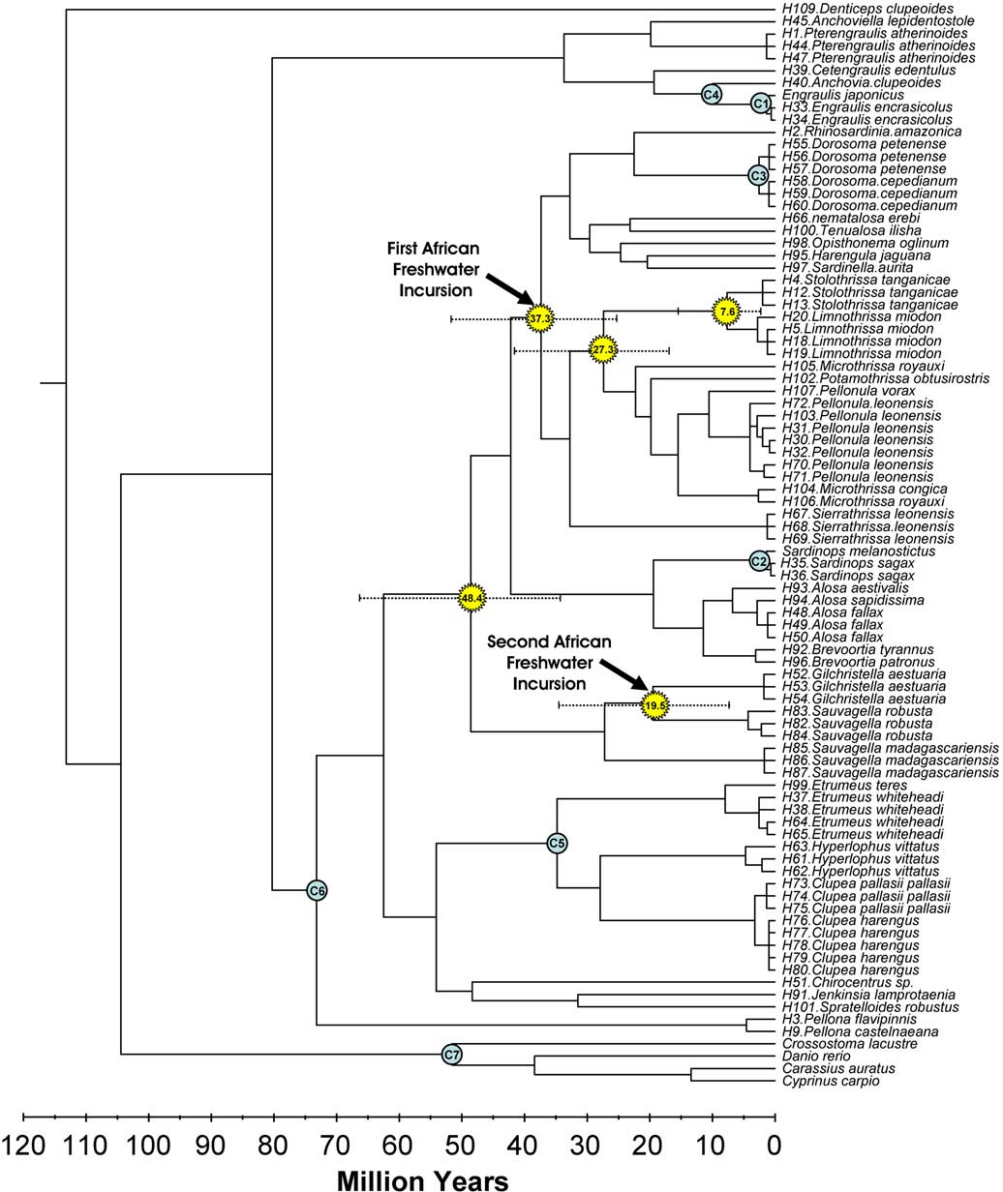
Linearized phylogenetic tree with node ages calculated with Multidivtime [64] using 12S, 16S and Cytochrome b gene partitions. Fossil calibration points (C1–C7), key divergence times and inferred freshwater colonization events are indicated on the phylogeny along with 95% reliability intervals.

Three independent runs of the relaxed clock generated consistent results (Fig. 3). Molecular clock calibrations indicate that pellonuline herring reached Madagascar 48 MYA (95% reliability interval: 34.0–66.2). Mainland Africa was subsequently colonized twice by pellonulids: (1) an independent colonization of western Africa by the Pellonulini approximately 37 MYA (95% reliability interval: 25.0–53.3 MYA) and (2) the southern Africa colonization of *Gilchristella aestuaria* from Malagasy ancestors which took place 20 MYA (95% reliability interval: 7.5–34.4 MYA) (Fig. 3). The pellonuline herring of Lake Tanganyika diverged from a large group of West African species approximately 27 MYA (95% reliability interval: 25.0–53.3 MYA), diverging into the two present-day Tanganyikan endemics 8 MYA (95% reliability interval: 2.1–15.9 MYA). While the reliability intervals of these divergence time estimates are large, they are consistent with major geophysical changes on the African continent. The colonization of West Africa 37 MYA is consistent with the end of a major marine incursion in the region (Fig. 1) and the split between the two Tanganyikan endemics suggests divergence during the early stages of lake formation approximately 9–12 MYA.

## Discussion

The endemic herring of Lake Tanganyika are the descendants of a group of herring that colonized the African continent during a major marine incursion that occurred in West Africa from 100-35 MYA. Pellonuline herring subsequently diversified in West Africa, spreading across the continent and reaching Lake Tanganyika during the early stages of its formation. While Lake Tanganyika was never in direct contact with the ocean, the herring of the lake are the first group whose ancestry can be traced back to a marine environment, indirectly supporting Moore's [16] thesis on the marine affinities of Tanganyika's biota. The herring of Lake Tanganyika have not diverged significantly from their West African relatives in morphology [19], indicating that the exceptional stability of the Lake has not prompted dramatic morphological innovation in this group, a hypothesis which has been put forward to explain the diversity of other Lake inhabitants [14].

In the absence of close relatives for most of the thalassoid taxa of Lake Tanganyika, it remains difficult to determine what proportion of the morphological diversity found in the Lake is due to *in situ* diversification and how much of this diversity reflects characteristics already present in the Lake's colonizers. Recent work suggests that the gastropods of Lake Tanganyika may be an interesting candidate for research in this respect [27]. While early researchers including Moore [16] were unable to identify close relatives of Tanganyika's gastropods outside the lake, a recent study has suggested that at least four extant genera of snails may be close relatives of the major lineages of Tanganyikan gastropods [12], [27]. Three of four of these genera are restricted to the Congo basin and West Africa, while the fourth is widespread in Africa, Madagascar and the Middle East. Molecular analyses confirm a close genetic relationship between this widespread lineage (*Cleopatra* spp.) and a group of Tanganyikan endemics [12], [28], but the remaining three genera have not yet been the focus of a phylogenetic study. The characterization of these outgroup gastropod taxa would allow the determination of the timing of the colonization and diversification of this group in Lake Tanganyika and would help to clarify whether the pattern of freshwater colonization and spread exhibited by pellonuline herring is relevant for other taxonomic groups.

### Geological Evidence Suggests Repeated Marine Incursions Into Central Africa

Moore [16] identified vast marine deposits in the African interior and used this finding as one of his strongest arguments for a historical connection between Lake Tanganyika and the sea. More recent geophysical surveys indicate that major marine incursions into Africa have occurred repeatedly in geological time. Fossils of marine fishes have been found in limestone beds of the Congo basin, providing conclusive evidence of a major marine incursion during the late Jurassic (150 MYA) [29]. Analyses of the sedimentology of Central and West Africa indicate that sea level increases caused by climate fluctuations continued to spur marine incursions after the Jurassic and a large marine seaway is thought to have extended from Libya to West Africa from the late Cretaceous through to the Eocene [Fig. 1; 23]. Given the frequency and extent of marine incursions into the African continent over the past 150 MY, it is somewhat surprising that so little attention has been paid to the possibility that freshwater capture of marine organisms has contributed to the present-day aquatic biodiversity of Africa.

While the frequency of marine incursions into the Congo basin is thought to have slowed after the Mesozoic due to changes in the geology of the region [29], Beadle [30] indicates that the Congo basin was dominated by a large inland sea during the Pliocene (2–5 MYA). If this is indeed the case, the presence of a large stable water body in central Africa at this time may have facilitated the dispersal of freshwater organisms between western and eastern Africa. The presence of this palaeolake may have also fostered increased rates of speciation during the Pliocene, a pattern recently suggested for the cichlid fishes of palaeolake Makgadikgadi in southern Africa [31].

### Lake Tanganyikan Herring: Evolutionary Stasis Despite Early Colonization

Molecular clock estimates indicate that the herring of Lake Tanganyika have been present in the lake for at least 2 MY and likely much longer (7.6MY; 95% reliability interval: 2.1–15.9MY). However, despite an extended tenure in the lake, this group has only diversified into two species, an extremely modest diversity when compared to the more than 200 species of cichlid fishes found in Lake Tanganyika [11]. Cichlid fishes have radiated repeatedly, both in the neotropics and in Africa, most notably in the African Great Lakes, where lineages of at least 200, 700 and 500 cichlid fishes are found in Lakes Tanganyika, Malawi and Victoria respectively [32]. Several recent reviews of evolutionary radiations have identified three key stages which characterize species-rich radiations [3], [33]. The first stage, diversification in habitat, occurs in the early stages of evolutionary radiations, when resource competition promotes the use of different habitats. The second phase of evolutionary radiation involves further niche partitioning, as morphological changes allow species to exploit underutilized resources. Streelman and Danley [3] suggest that species diversity can only be fully realized after a third phase of radiation, diversification in secondary sexual characteristics associated with reproduction. While the order and importance of these three stages may vary among radiations, most species-rich radiations appear to have involved some form of all three of these stages. While there is some degree of habitat partitioning between the more onshore (*Limnothrissa miodon*) and offshore (*Stolothrissa tanganicae*) herring species of Lake Tanganyika [22], these species have not significantly diverged in their resource utilization [34] and there is no indication of secondary sexual characteristics associated with assortative mating, suggesting that any radiation of this group is still in its initial phase. The lack of major radiation of the herring of Tanganyika may be due to intrinsic differences between herring and cichlid fishes which influence their speciation potential [33] or may be related to the different habitats inhabited by these species. Alternatively, the potential for an adaptive radiation of herring may have been limited by the presence of an already diverse cichlid fauna in the lake soon after its formation (see above).

As essentially pelagic fishes, the possibility for allopatric divergence in the herring of Lake Tanganyika may be reduced when compared to the nearshore cichlids of the Lake. Several recent studies have highlighted the importance of lake level fluctuations in the diversification of its endemic cichlids [35], [36]. These authors suggest that allopatric speciation likely played an important role in the initial stages of diversification among littoral cichlids. The sole population genetic study of Tanganyika's herring revealed no significant population structure in populations of *L. miodon* from the lake [37], indicating little evidence of intralacustrine divergence in this species. The species-level diversity of pelagic cichlids of Lake Tanganyika is also lower than that of littoral groups [38], though modest radiations have occurred in several tribes of pelagic and deep-water cichlids [39]–[41].

### Clupeiform Fishes: Weak Support for Traditional Subfamily Relationships

While the taxonomic sampling here is the most comprehensive of any molecular study of clupeomorph fishes, several groups are nonetheless only poorly represented (<20% of Pristigasteridae, Engraulidae, Clupeinae and Dorostomatinae; Table 2). Grande [21] suggested that the Clupeinae, Alosinae and Dorostomatinae were likely all artificial groupings that would be further subdivided following further investigations. This hypothesis is supported for the Clupeinae (4 distinct lineages and statistical rejection of subfamily monophyly), but monophyly of both the Alosinae and Dorostomatinae cannot be statistically rejected. As only a subset of species from each of these subfamilies were included here (Table 2), future studies should aim to exhaustively sample species at the subfamily level to rigorously test Grande's morphological hypotheses.

**Table 2:** Taxonomic sampling of the present study.

Order	Suborder	Superfamily	Family	Subfamily	Extant Species	Included Species
Clupeiformes						
	Denticipitoidei				1	1
	Clupeoidei					
		Pristigasteroidea	Pristigasteridae	Pristigasterinae	30	2
		Engrauloidea	Engraulidae		130	6
		Clupeoidea	Chirocentridae		2	1
			Clupeidae	Pellonulinae	41	12
				Dussumieriinae	11	4
				Dorostomatinae	22	3
				Alosinae	19	5
				Clupeinae	61	9

Clupeiform taxonomic groupings follow Grande [21]. The endemic herring of Lake Tanganyika (*Stolothrissa tanganicae* and *Limnothrissa miodon*) are members of the Pellonulinae.

Although almost 2000bp of sequence data were analyzed for the taxa included here, phylogenetic relationships at deeper branches in the phylogeny remain only poorly resolved. These results are consistent with two recent investigations of clupeiform fishes which, despite similar taxonomic sampling, yielded conflicting results concerning several of the intraorder relationships. Li and Orti [42] employed a combination of mitochondrial and nuclear genes to investigate relationships among the Clupeiformes. Li and Orti statistically rejected the monophyly of the Clupeinae and found that *Denticeps clupeoides* clustered together with the cyprinid outgroups included in their study, a pattern that they suggested might be due to the high GC content of this species relative to other clupeomorphs.

A second recent study used complete mitochondrial genome sequences to investigate the clupeiform question [43]. In contrast with the results of Li and Orti, Lavoue *et al*. found that *Denticeps clupeoides* clustered together with the other Clupeomorphs. This study also statistically rejected the monophyly of the Clupeidae as well as the subfamilies Alosinae, Clupeinae and Dorostomatinae, in line with Grande's [21] hypothesis of polyphyly of these groups on the basis of morphological data. Both Lavoue *et al* and Li and Orti supported a sister-group relationship between the Engrauloidea and Clupeoidea [44], a pattern also found here, but the two analyses conflict in their placement of the Pristigasteridae and Chirocentridae, two groups whose placement is also only weakly supported in this study. While the results of the Li and Orti [42] and Lavoue *et al.* [43] studies suggest that additional molecular data might help to better resolve relationships among the clupeiform fishes, more extensive taxonomic sampling will be essential before undertaking a major revision of this group.

Of particular interest in light of the marine incursion scenario put forth here is the grouping of *Ethmalosa fimbriata*, an estuarine species widespread along the coasts of West Africa, with the freshwater pellonuline herring found in the region [43]. This species has been the focus of a recent phylogeographic study [45], which suggests that the historical population structure of the species has been strongly influenced by Pleistocene sea level fluctuations in the region, when local populations of the species were isolated in freshwater refuges. As this euryhaline species may be the closest living marine relative of the freshwater pellonulines of West Africa, future comparisons between the morphology and physiology of *Ethmalosa* and its pellonuline relatives may help to identify key innovations that allowed the ancestor of these groups to successfully colonize freshwater.

## Materials and Methods

### Sample Collection, PCR Amplification and DNA Sequencing

Specimens were collected by the authors or provided by colleagues between 1999–2003 (Tables 2 & 3). All specimens were preserved in 70% ethanol and total genomic DNA was extracted by proteinase K/SDS digestion and purified by phenol-chloroform extraction and ethanol precipitation [46]. Several recent investigations have supported a close phylogenetic relationship between clupeiform fishes and the Ostariophysi [47], [48]. Published sequences for *Carassius auratus, Crossostoma lacustre, Cyprinus carpio,* and *Danio rerio* (Cypriniformes) as well as *Gonorhynchus greyi* and *Chanos chanos* (Gonorhynchiformes) were included as outgroups.

**Table 3:** Specimen collection information.

Sample #	Species	Collection Locality (Country) (Date)	Collector/Reference
**H1**	*Pterengraulis atherinoides*	Braganca Paulista (Brazil) (16/07/00)	AM
**H2**	*Rhinosardinia amazonica*	Braganca Paulista (Brazil) (16/07/00)	AM
**H3**	*Pellona flavipinnis*	(Brazil)	AM
**H4**	*Stolothrissa tanganicae*	Lake Tanganyika (Zambia) (03/11/99)	ABW
**H5**	*Limnothrissa miodon*	Lake Tanganyika (Zambia) (03/11/99)	ABW
**H9**	*Pellona castelnaeana*	(Brazil)	IF
**H12**	*Stolothrissa tanganicae*	Lake Tanganyika (Zambia) (25/12/00)	ABW
**H13**	*Stolothrissa tanganicae*	Lake Tanganyika (Zambia) (25/12/00)	ABW
**H18**	*Limnothrissa miodon*	Malagarasi River (Tanzania) (12/12/00)	ABW
**H19**	*Limnothrissa miodon*	Malagarasi River (Tanzania) (12/12/00)	ABW
**H20**	*Limnothrissa miodon*	Malagarasi River (Tanzania) (12/12/00)	ABW
**H30**	*Pellonula leonensis*	Tano Basin (Ivory Coast) (XX/04/00)	GTT
**H31**	*Pellonula leonensis*	Tano Basin (Ivory Coast) (XX/04/00)	GTT
**H32**	*Pellonula leonensis*	Tano Basin (Ivory Coast) (XX/04/00)	GTT
**H33**	*Engraulis encrasicolus*	Hout Bay (South Africa) (08/08/01)	CVL
**H34**	*Engraulis encrasicolus*	Hout Bay (South Africa) (08/08/01)	CVL
**H35**	*Sardinops sagax ocellatus*	Hout Bay (South Africa) (07/08/01)	CVL
**H36**	*Sardinops sagax ocellatus*	Hout Bay (South Africa) (07/08/01)	CVL
**H37**	*Etrumeus whiteheadi*	Hout Bay (South Africa) (07/08/01)	CVL
**H38**	*Etrumeus whiteheadi*	Hout Bay (South Africa) (07/08/01)	CVL
**H39**	*Cetengraulis edentulus*	Braganca (Brazil) (1999)	UK
**H40**	*Anchovia clupeoides*	Braganca (Brazil) (1999)	UK
**H44**	*Pterengraulis atherinoides*	Braganca (Brazil) (1999)	UK
**H45**	*Anchoviella lepidentostole*	Braganca (Brazil) (1999)	UK
**H47**	*Pterengraulis atherinoides*	Braganca (Brazil) (1999)	UK
**H48**	*Alosa fallax (Severn33)*	Severn (England) (June 1–6/00)	MA
**H49**	*Alosa fallax (Severn40)*	Severn (England) (June 1–6/00)	MA
**H50**	*Alosa fallax (Severn44)*	Severn (England) (June 1–6/00)	MA
**H51**	*Chirocentrus sp.*	(Singapore) (XX/XX/98)	BV
**H52**	*Gilchristella aestuaria*	Eastern Cape (South Africa) (09/11/01)	RB
**H53**	*Gilchristella aestuaria*	Orange River (Nigeria) (02/05/01)	RB
**H54**	*Gilchristella aestuaria*	Lake Piti (Mozambique) (29/09/01)	RB
**H55**	*Dorosoma petenense*	Brazos River (Texas) (04/02/02)	RL
**H56**	*Dorosoma petenense*	Brazos River (Texas) (04/02/02)	RL
**H57**	*Dorosoma petenense*	Brazos River (Texas) (04/02/02)	RL
**H58**	*Dorosoma cepedianum*	Lake Wauberg (Florida) (22/01/02)	KT
**H59**	*Dorosoma cepedianum*	Lake Wauberg (Florida) (22/01/02)	KT
**H60**	*Dorosoma cepedianum*	Lake Wauberg (Florida) (22/01/02)	KT
**H61**	*Hyperlophus vittatus*	Bunbury, Western Australia (Australia) (XX/01/02)	DG
**H62**	*Hyperlophus vittatus*	Bunbury, Western Australia (Australia) (XX/01/02)	DG
**H63**	*Hyperlophus vittatus*	Bunbury, Western Australia) (Australia) (XX/01/02)	DG
**H64**	*Etrumeus whiteheadi*	Hout Bay (South Africa) (07/08/01)	CVL
**H65**	*Etrumeus whiteheadi*	Hout Bay (South Africa) (07/08/01)	CVL
**H66**	*Nematalosa erebi*	Fish River, Darwin (Australia) (05/09/01)	HL
**H67**	*Sierrathrissa leonensis*	Volta Basin (Ghana) (23/01/01)	GTT
**H68**	*Sierrathrissa leonensis*	Volta Basin (Ghana) (23/01/01)	CVL
**H69**	*Sierrathrissa leonensis*	Volta Basin (Ghana) (23/01/01)	CVL
**H70**	*Pellonula leonensis*	Volta Basin (Ghana) (23/01/01)	CVL
**H71**	*Pellonula leonensis*	Volta Basin (Ghana) (23/01/01)	CVL
**H72**	*Pellonula leonensis*	Volta Basin (Ghana) (23/01/01)	CVL
**H73**	*Clupea pallasii pallasii*	Cape Flattery, Washington (USA) (XX/09/02)	LW
**H74**	*Clupea pallasii pallasii*	Cape Flattery, Washington (USA) (XX/09/02)	LW
**H75**	*Clupea pallasii pallasii*	Cape Flattery, Washington (USA) (XX/09/02)	LW
**H76**	*Clupea harengus*	Sept Iles, Quebec (Canada) (17/06/02)	IM
**H77**	*Clupea harengus*	Sept Iles, Quebec (Canada) (17/06/02)	IM
**H78**	*Clupea harengus*	Sept Iles, Quebec (Canada) (17/06/02)	IM
**H79**	*Clupea harengus*	La Romaine, Quebec (Canada) (07/06/02)	IM
**H80**	*Clupea harengus*	La Romaine, Quebec (Canada) (07/06/02)	IM
**H82**	*Sauvagella robusta*	Ambomboa River (Madagascar) (XX/XX/96)	JS
**H83**	*Sauvagella robusta*	Ambomboa River (Madagascar) (XX/XX/96)	JS
**H84**	*Sauvagella robusta*	Ambomboa River (Madagascar) (XX/XX/96)	JS
**H85**	*Sauvagella madagascariensis*	Onive River (Madagascar) (XX/02/94)	JS
**H86**	*Sauvagella madagascariensis*	Onive River (Madagascar) (XX/02/94)	JS
**H87**	*Sauvagella madagascariensis*	Onive River (Madagascar) (XX/02/94)	JS
**H91**	*Jenkinsia lamprotaenia*	Carrie Bow Bay (Belize) (07/19/91)	EW
**H92**	*Brevoortia tyrannus*	mid Atlantic Bight (USA) (03/09/95)	KS
**H93**	*Alosa aestivalis*	mid Atlantic Bight (USA) (03/09/95)	KS
**H94**	*Alosa sapidissima*	mid Atlantic Bight (USA) (03/09/95)	KS
**H95**	*Harengula jaguana*	Brownsville, Texas (USA) (06/19/02)	KM
**H96**	*Brevoortia patronus*	Brownsville, Texas (USA) (06/19/02)	KM
**H97**	*Sardinella aurita*	Brownsville, Texas (USA) (06/19/02)	KM
**H98**	*Opisthonema oglinum*	Brownsville, Texas (USA) (06/19/02)	KM
**H99**	*Etrumeus teres*	Brownsville, Texas (USA) (06/19/02)	KM
**H100**	*Tenualosa ilisha*	Padma River (Bangladesh) (15/01/04)	HK
**H101**	*Spratelloides robustus*	Myponga, Gulf St. Vincent (Australia) (29/04/01)	PR
**H102**	*Potamothrissa obtusirostris*	Congo River, Brazzaville (16/01/03)	VM
**H103**	*Pellonula leonensis*	Gamba Lagoon, Brazzavlle (Congo-Brazzaville) (10/02/03)	VM
**H104**	*Microthrissa congica*	Congo River, Brazzaville (Congo-Brazzaville) (16/01/03)	VM
**H105**	*Microthrissa royauxi*	Congo River, Brazzaville (Congo-Brazzaville) (16/01/03)	VM
**H106**	*Microthrissa congica*	Congo River, Malebo (Congo-Brazzaville) (28/05/03)	VM
**H107**	*Pellonula vorax*	Ndogo Lagoon (Congo) (10/02/03)	VM
**H109**	*Denticeps clupeoides*		AM
**NC_003097**	*Engraulis japonicus*		[76]
**NC_002616**	*Sardinops melanostictus*	(Japan)	[77]
**NC_004702**	*Gonorhynchus greyi*	(Australia)	[78]
**NC_004693**	*Chanos chanos*	Sulawesi (Indonesia)	[78]
**NC_002079**	*Carassius auratus*		[79]
**NC_001727**	*Crossostoma lacustre*	Dahu River, Taiwan (China)	[80]
**NC_001606**	*Cyprinus carpio*		[81]
**NC_002333**	*Danio rerio*		[82]

Collectors: ABW (Tony Wilson); AM (Axel Meyer); BV (Byrappa Venkatesh); CVL (Carl van der Lingen/Megan Terry); DG (Daniel Gaughan); EW (Ed Wiley); GTT (Guy Teugels); HL (Helen Larson); HK (Haseena Khan); IF (Izeni Farias); IM (Ian McQuinn); JS (John Sparks/Melanie Stiassny); KM (Kris McNyset); KS (Kate Shaw); KT (Kim Tugend/Mike Allen); LW (Laurie Weitcamp/Mike Ford); MA (Miran Aprahamian); PR (Paul Rodgers); RB (Roger Bills/Sally Terry); RL (Raymond Li/Fran Gelwick); UK (Uwe Krumme), VM (Victor Mamonekene/Melanie Stiassny).

The polymerase chain reaction (PCR) was used to amplify a total of 2,608 bp from three fragments of mitochondrial DNA. A 548 bp segment of the large subunit (16S) mitochondrial ribosomal gene was amplified using primers L2510 and H3058 [49], while primers L1090 [46] and H2001 [50] were used to amplify 911 bp of the small subunit (12S) mitochondrial ribosomal gene, tRNA-Valine and 16S. 1,149 bp of the cytochrome *b* (Cyt*b*) gene were amplified with L14725 [51] and H15926 [52]. Reaction conditions are described in Wilson et al. [52]. Sequencing reactions were prepared as in Wilson *et al.* [52] and visualized on an ABI 3100 automated sequencer. DNA sequences have been submitted to GenBank (Accession numbers: EU552549-EU552793).

### Sequence Alignment and Phylogenetic Reconstruction

The orthologous DNA sequences obtained were aligned, using default settings, by CLUSTALW [53] and optimized by eye. Optimization of rDNA gene fragment alignments was facilitated through the use of secondary structure models for teleost long and short subunit RNAs [54], [55]. Regions of the optimized alignment which could not be reliably aligned were eliminated from analysis (data alignment available upon request), resulting in an alignment of 525 bp for 16S, 520 bp for 12S and 1,149 bp for the Cyt*b* dataset, for a total of 2,194 bp. Data partitions were tested for substitution saturation using a non-parametric statistical test implemented by DAMBE 4.5.47 [56]. Prior to concatenating the three sequence alignments, the congruency of data partitions was tested with a likelihood-based congruency test (α = 0.05; 10000 RELL bootstrap replicates) [57], using maximum likelihood (ML) topologies generated from individual gene analyses as well as the overall ML tree (see below).

Neighbor-joining distance and maximum parsimony analyses were performed with PAUPV4b10 [58], with indels coded as missing data. Parsimony minimal analyses included a full heuristic search with random addition (50 replicates), the TBR branch swapping algorithm and the MULPARS option. For parsimony analyses, a transversion/transition weighting of three was used. Neighbor-joining analyses applied a GTR+I+G model of substitution [59], with transition rate matrix (1.9150 9.8250 3.6271 0.8214 17.2997), gamma shape parameter (0.5214), proportion of invariable sites (0.4838) and nucleotide frequencies (A: 0.2764; C: 0.2780; G: 0.2168; T: 0.2288) estimated from the dataset using Modeltest V3.7 [60]. Reliability of phylogenetic signal was tested using 500 bootstrap replicates for both parsimony and NJ distance analyses. A single random addition of taxa was used for each replicate of the parsimony bootstrap.

The overall ML tree topology for each gene and the concatenated dataset was determined using GarliV0.951 [61] with model parameters as estimated by Modeltest. The initial tree topology was constructed by random addition, the stopgen and stoptime parameters were both set to 10,000,000 and search termination settings were set at default values. Four independent runs of each tree search produced final likelihood values that varied by less than 3.5. The tree was the highest likelihood value was used for subsequent analyses. Phylogenetic reliability of the overall ML tree was tested using 500 bootstrap replicates.

Phylogenetic relationships were also estimated according to a Bayesian method of phylogenetic inference implemented by MrBayes v3.1.2 [62]. Posterior probabilities of phylogenetic trees were approximated by a 1,000,000-generation Metropolis-coupled Markov chain Monte Carlo simulation (MCMCMC; four chains, chain temperature = 0.2), under a GTR+I+G model of sequence evolution, with simultaneous estimation of parameters, sampling every 1,000th generation. A 50% majority-rule consensus tree was constructed following a 100,000-generation burn-in to allow chains to reach stationarity. Three separate runs of MrBayes v3.1.2 under these parameter settings generated qualitatively similar results.

To test morphological-based hypotheses on the taxonomic relationships among clupeiform fishes, the ML topology and branch lengths were recalculated as above, with major groupings constrained to be monophyletic. The deviation between these alternative topologies and the unconstrained ML topology was tested using a Shimodaira-Hasegawa (SH) test [63] with 10000 RELL bootstrap replicates.

### Fossil Calibration and Molecular Clock

To investigate whether rates of molecular evolution fit with a strict molecular clock model, the likelihood of the ML phylogeny was recalculated with the constraint of global molecular clock using the Rambaut parameterization for clock optimization implemented in PAUP4b10 [58]. The likelihood of the clock-based tree was compared with that of the unconstrained topology using a likelihood ratio test (LRT).

A relaxed molecular clock method allowing autocorrelated rates of evolution along branches [64] was also implemented here. This Bayesian-based method allows for uncertainty in fossil calibration points and permits variation in rates of molecular evolution among genes. Molecular clock calibration followed the protocols outlined in Rutschmann [65]. Briefly, model parameters were estimated for each gene partition using PAML V3.14 [66] under a model of evolution incorporating variable nucleotide frequencies, a transition:transversion parameter and nucleotide variation across sites [F84+G model; described in 67]. Branch lengths of the ML tree were optimized for each gene partition and the variance-covariance matrix of evolutionary rates was estimated using Estbranches. Finally, divergence time estimates were calculated using the Bayesian MCMCMC approach implemented in Multidivtime [64], which simultaneously considers branch length estimates and variance-covariance matrices from each data partition. Posterior probabilities of divergence time estimates were determined following a 100,000 cycle burn-in. The MCMCMC chain was sampled every 100^th^ cycle for a total of 2,000,000 cycles. Rates of genetic change were set to vary freely among gene partitions and a prior root-to-tip divergence time estimate was set at 146 MY. Three runs of this program from different starting points yielded consistent estimates of divergence times.

A suite of seven fossil calibration points for clupeoid and cyprinid fishes were included for calibration of the molecular clock used here: C1–Earliest fossil of *Engaulis japonicus*: 0–2 MY (Kokubu Group, Japan; Yabumoto [68]), C2–Earliest fossil of *Sardinops melanostictus*: 0–2 MY (Kokobu Group, Japan; Yabumoto [68]), C3–Earliest fossil of *Dorosoma petenense*: 2–3 MY (Gatuna Formation, New Mexico; Miller [69]), C4–Earliest engraulid fossil: *Engraulis tethensis*: 6–12 MY (Mesaoria Group, Cyprus; Grande and Nelson [70]), C5–Earliest *Etrumeus* sp. fossil: *Etrumeus hafizi*: 23–38 MY (Estabanhat, Iran; Arambourg [71], Grande [21]), C6–Earliest pristigasterid fossil: *Gastroclupea branisai*: 66–94 MY (El Molino Formation, Bolivia; Branisa [72], Grande [21]) and C7–Earliest cyprinid fossil: *Parabarbus* sp.: 49–55 MY [Sytchevskaya (1986) in 73].
